# Novel Closing Method Using Subcutaneous Continuous Drain for Preventing Surgical Site Infections in Radical Cystectomy

**DOI:** 10.1155/2014/897451

**Published:** 2014-03-10

**Authors:** Yasuhiko Hirose, Taku Naiki, Ryosuke Ando, Akihiro Nakane, Toshiki Etani, Keitaro Iida, Hidetoshi Akita, Takehiko Okamura, Kenjiro Kohri

**Affiliations:** ^1^Department of Nephro-Urology, Nagoya City University Graduate School of Medical Sciences, 1 Kawasumi, Mizuho-Cho, Mizuho-Ku, Nagoya 467-8601, Japan; ^2^Department of Urology, J. A. Aichi Anjo Kosei Hospital, 28 Higashihirokute, Anjo-Cho, Anjo 446-0082, Japan

## Abstract

To reduce the incidence of surgical site infection (SSI) after radical cystectomy, a new closing method using subcutaneous continuous aspiration drain was developed and compared to the conventional closing method. The new method involved (a) closed aspiration with an indwelling aspiration drain without suture of the subcutaneous fat and (b) covering with hydrocolloid wound dressing after suture of the dermis with 4-0 absorbable thread and reinforcement using strips. The incidence of SSI was significantly improved by using the new method. Furthermore, univariate and multivariate analysis associated with SSI revealed that the new closing method was statistically correlated with 85% reduction of SSI (odds ratio: 0.15, 95% confidence interval: 0.03–0.69).Our new method using continuous aspiration with subcutaneous drain is useful for preventing SSI through removal of effusions and reduction of dead space by apposition of the subcutaneous fat.

## 1. Introduction

Surgical site infection (SSI) continues to be one of the most common complications in conventional abdominal surgery, with the incidence of infected wounds after radical cystectomy ranging from 2.9 to 46.0% [1–5]. Although use of a subcutaneous continuous suction drainage system has been suggested to help prevent SSI, it is mainly used in the colorectal and gynecological fields [6–8], and the effects of prophylactic subcutaneous drainage are not well studied in radical cystectomy. Although we have practiced strict infection control with measures such as diligent hand washing before surgery, short-term administration of single antibiotics, application of dressings directly after surgery, and use of quick-drying alcoholic agents according to guidelines [9, 10], these measures have not resulted in sufficient control of SSI at our hospital. Therefore, in our hospital, from January 2010, we have changed the method of closing surgical wounds in radical cystectomy from the conventional method to a combination of subcuticular suture and continuous subcutaneous drain. The present investigation was performed to evaluate the efficacy of these interventions for the prevention of SSI.

## 2. Patients and Methods

**Figure 1:**
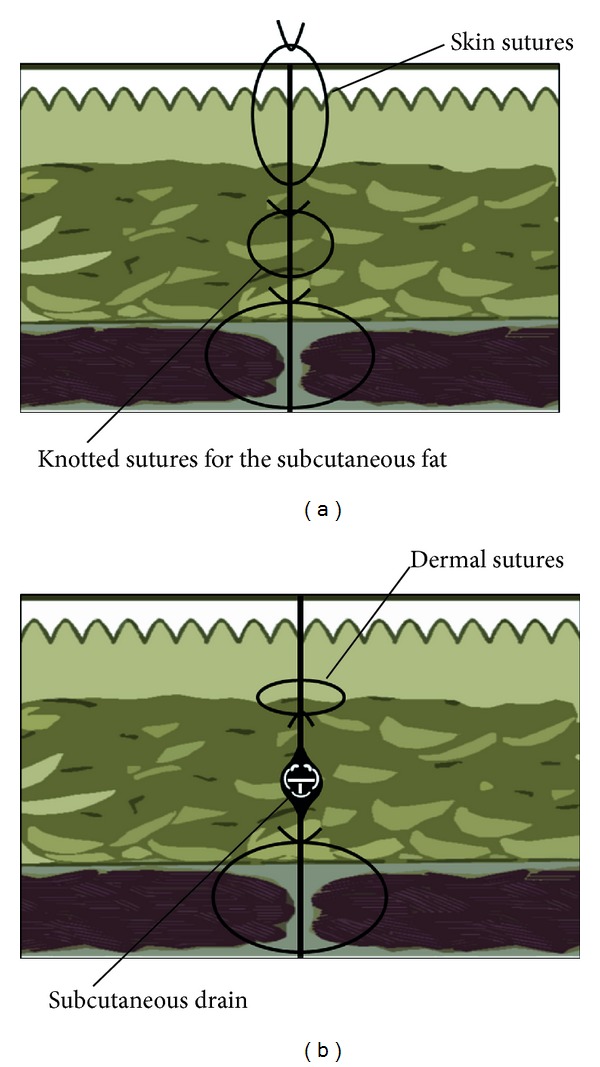
Method of wound closure analyzed in this study. (a) Conventional method from 2002 to 2009. (b) New method from 2010 to 2012.

**Figure 2:**
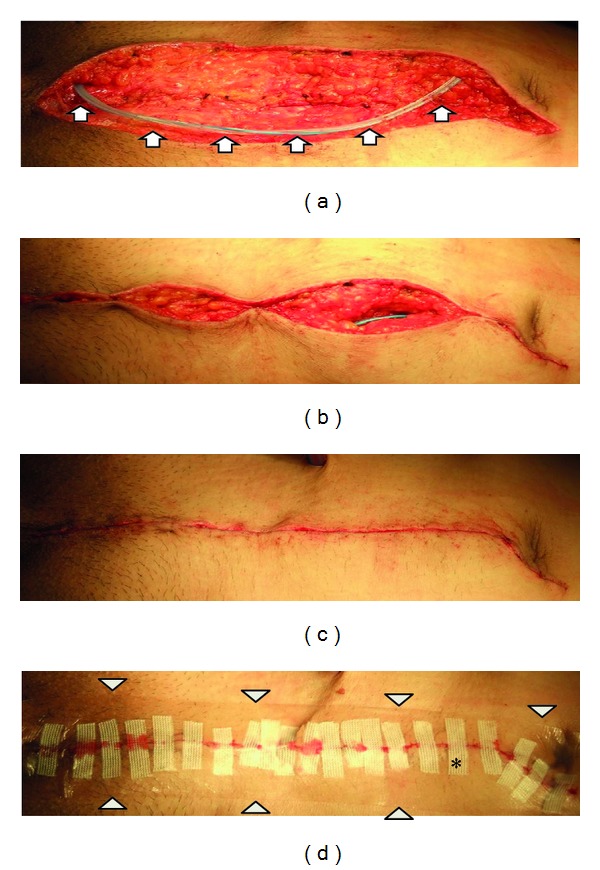
Procedure of the new method. (a) Aspiration drain was subcutaneously indwelled. Arrow: closed drain. ((b) and (c)) Dermal suture using 4-0 absorbable thread. (d) Covering with hydrocolloid wound dressing and reinforcement using strips. Trigonal dot: hydrocolloid dressing; asterisk: strips.

We retrospectively studied 90 patients who underwent radical cystectomy in the Department of Urology at Anjou Kosei Hospital of the Aichi Prefecture Welfare Association from 2002 to 2012. The subjects of this review were 63 patients with conventional surgical wound closure (conventional method), that is, knotted sutures of absorbable thread for the subcutaneous fat followed by knotted sutures of nylon thread, from 2002 to 2009 (Figure 1(a)) and 27 patients with closure by the new method as described below from January 2010 to 2012 (Figure 1(b)). The new method involved (a) closed aspiration with an indwelling aspiration drain (10Fr J-VAC Blake silicon drain: Ethicon, Somerville, NJ) without suture of the subcutaneous fat (Figures 2(a) and 2(b)) and (b) covering with hydrocolloid wound dressing (Karayahesive: ALCARE, Tokyo, Japan) after suture of the dermis with 4-0 absorbable thread and reinforcement using strips (Steri strips: sumitomo3M, Tokyo, Japan) (Figures 2(c) and 2(d)).

In all patients, clinical pathways were used, and cefotiam hexetil hydrochloride (second generation of cephem) was administered via an intravenous route for prophylactic antibiotic medication 30 min before initiation of surgery, followed by dripping of the antibiotics 3–6 h later. Antibiotics were given three times on the day of surgery and for three days after surgery. Radical cystectomy was performed with pelvic lymphadenectomy according to the standard procedures in both groups. Briefly, patients were placed in the supine position and a midline lower abdominal 15–20 cm incision was made. The method of diversion with either an ileal conduit or ileal neobladder was selected according to the patient's preference and medical reasons. All operations were performed in the standard aseptic manner. The wound was protected with Karayahesive for 7 days. The drain was maintained with continuous bulb suction and removed 48 h after surgery. The primary goal of this study was to investigate wound complication rates in both groups and, thus, to evaluate the efficacy of interventions for the prevention of SSI. The assessment of SSI was based on the Centers for Disease Control and Prevention (CDC) guideline [9]. SSI was defined as a wound with purulent drainage or symptoms (e.g., tenderness, erythema, and swelling) within 30 postoperative days. Incisional SSI at dermis or subcuticular tissue was referred to as superficial SSI, SSI at deep level of subcuticular tissue was referred to as deep SSI, and SSI in abdominal cavity or organ was referred to as organ/space SSI. Patient characteristics and variables included age, gender, body mass index, thickness of subcutaneous fat, prevalence of diabetes mellitus, smoking habit, length of operation, intraoperative blood loss, difference in urinary diversion, and method of wound closure. Univariate and multivariate statistical analyses for risk factor of SSI were conducted for all 90 patients who underwent radical cystectomy. This study was conducted in accordance with the Declaration of Helsinki. All patients were fully informed of the disease, operative procedures, and complications and were required to sign a written informed consent form. The operative procedures were approved by the ethics committee of Anjo Kosei Hospital.

## 3. Statistical Analyses

The demographic variables were calculated and tabulated to compare between patients who underwent conventional and new closing methods. Categorical variables were compared using the chi-square test or Fisher's exact test, and mean values of continuous variables were compared between groups using Student's or Welch's *t*-test. Logistic regression analysis was used to estimate the odds ratio (OR) and 95% confidence intervals (CI) for the incidence of SSI after radical cystectomy. All the statistical analyses were performed for crude as well as age- and gender-adjusted models. All statistical analyses were performed using the Statistical Analysis system, version 9.3 (SAS Institute, Cary, NC), and significance was defined as *P* < 0.05.

## 4. Results

The clinical characteristics of all entry patients are listed in Table 1. There were no significant differences in the risk factors for SSI between the conventional method and new method groups, namely, in age, gender, body mass index, thickness of subcutaneous fat, prevalence of diabetes mellitus, smoking habit, operation time, amount of bleeding, difference in urinary diversion, and method of wound closure (Table 1). Of the 63 patients who underwent the conventional method, the incidence of SSI was 34.9% (22 patients), whereas the incidence was 7.4% (2 of 27 patients) in patients who underwent the new method (Table 1). Risk factors for development of SSI were evaluated by univariate and multivariate statistical analyses. Ten variables were used for analyses, and the conventional method was found to be the only risk factor of SSI. Compared to the conventional method, the OR of the new method for SSI was 0.15 (95% CI: 0.03–0.69) by univariate analysis and 0.14 (95% CI: 0.03–0.69) by multivariate analysis (Table 2).

## 5. Discussion

Radical cystectomy is the treatment of choice for patients with invasive bladder cancer. The surgical routine of this procedure has improved and even less invasive laparoscopic techniques can now be applied; nonetheless, it remains a procedure with significant morbidity and potentially life-threatening complications. The morbidity of radical cystectomy is clearly lower than in previous decades; however, one of the common complications is SSI [11, 12]. The occurrence of SSI leads to longer hospital stays after surgery, and the quality of life of the patients is diminished. In addition, SSI is correlated with the escalation of health expenditure [13]; therefore, the reduction of SSI after radical cystectomy is an important clinical and economical issue.

Subcutaneous drain removes effusions and reduces dead space. The beneficial effect of subcutaneous drains has also been shown in some studies [6, 7], although in others there was no beneficial effect [14, 15]. In addition, the closing procedures besides using subcutaneous drainage system were various. We established a new method by combining subcutaneous drain and dermal suture, including hydrocolloid wound dressing, and obtained good results. In addition, Blake silicon drain is made of soft fluted silicone and has a wide surface area with four channels along the drain. Wide suction area results in low suction and pressure which may minimize damage to subcutaneous fat. According to the CDC guideline, it is recommended that removal of the drain should be performed as soon as possible. However, there is no consensus concerning the time of removal of subcutaneous drain. We removed the subcutaneous drain 48 h after operation, and as a result only 7.4% patients had SSI. It is difficult to define whether a single procedure could primarily contribute to a decrease in SSI rate; however, all of these factors may contribute to better patient outcome.

Various risk factors for incisional SSI have been reported, including obesity [8, 15, 16]. However, obesity (BMI ≥ 25) was not found to be a risk factor for SSI in our study. Subcutaneous fatty tissue is known to accumulate more in the infraumbilical area than in other abdominal regions, leading some gynecologists to accept the thickness of the subcutaneous fat, rather than BMI [17, 18]. However, the effect of obliteration of subcutaneous dead space in patients with tissue thickness >20 mm on prevention of SSI is controversial. In our study, subcutaneous tissue thickness was not demonstrated to be a risk factor. Therefore, further investigation is needed to clarify this.

SSI is among the leading causes. Therefore, based on these findings, the Dutch hospital patient safety program (DHPSP) was developed [19]. The DHPSP included a bundle to prevent the development of SSI, and the effect of improved discipline is now generally recognized as an important aspect [12, 20, 21]. This study was basically conducted in a retrospective manner; however, new methods of management of surgical wounds have been involved in the decrease in SSI incidence, as described above. By modifying our prevention method through the use of prophylaxis according to the DHPSP and timing of drain removal, we would like to further reduce the incidence of SSI after radical cystectomy.

## 6. Conclusion

In this study, we developed a new method using continuous aspiration with subcutaneous drain and closure of surgical wounds with dermal sutures and demonstrated that it is useful for preventing SSI through removal of effusions and reduction of dead space by apposition of the subcutaneous fat.

## Figures and Tables

**Table 1:** Patients' characteristics and incidence of SSI between the two groups.

Characteristics *n* = 90	Conventional method *n* = 63	New method *n* = 27	P*
Age (yr), mean ± SD	67.9 ± 9.5	68.4 ± 11.0	0.81
BMI (kg/m^2^), mean ± SD	23.3 ± 3.5	23.0 ± 3.4	0.74
Op. time (min), mean ± SD	470.8 ± 111.3	472.3 ± 93.0	0.95
Blood loss (mL), mean ± SD	2995.3 ± 2055.8	2364.3 ± 1183.2	0.07
Subcutaneous fat thickness (mm), mean ± SD	17.9 ± 6.8	17.9 ± 8.6	0.97

Male patients, *n* (%)	51 (81.0)	20 (74.1)	0.46
Over weight and obese (BMI ≥ 25), *n* (%)	22 (34.9)	7 (25.9)	0.40
Diabetes mellitus, *n* (%)	14 (22.2)	2 (7.4)	0.13
Smoking, *n* (%)	41 (65.1)	15 (55.6)	0.39
Urinary diversion			0.89
Cutaneostomy, *n* (%)	29 (46.0)	12 (44.4)	
Bowel-utilizing diversion, *n* (%)	34 (54.0)	15 (55.6)	
SSI, *n* (%)	22 (34.9)	2 (7.4)	<0.01
Superficial	5 (7.9)	0 (0.0)	
Deep	14 (22.2)	2 (7.4)	
Organ/space	3 (4.8)	0 (0.0)	

SSI: surgical site infection; BMI: body mass index; Op. time: operation time

*P value by *t*-test, chi-square test, or Fisher's exact test.

**Table 2:** Univariate and multivariate adjusted OR (95% CI) for SSI.

Risk factors for SSI	Univariate model		Multivariate model	
	OR	95% CI	OR	95% CI
Age (per 10 years)	0.82	0.51–1.32	—	—
Male patients	1.85	0.63–5.46	—	—
Subcutaneous fat thickness (>20 mm)	1.38	0.52–3.67	1.06	0.35–3.19
Over weight and obese (BMI ≥ 25)	2.26	0.86–5.94	1.97	0.70–5.55
Diabetes mellitus	2.61	0.85–8.05	2.83	0.89–8.98
Smoking	1.30	0.49–3.47	1.78	0.58–9.90
Op. time (>600 min)	2.44	0.60–10.0	2.39	0.58–9.90
Blood loss (>1500 mL)	1.60	0.48–5.38	1.39	0.40–4.84
Cutaneostomy	1.02	0.40–2.59	1.10	0.41–2.95
New closing method	0.15	0.03–0.69	0.14	0.03–0.64

OR: odds ratio; CI: confidence interval; SSI: surgical site infection; BMI: body mass index; Op. time: operation time.

Multivariate model: adjusted for age and gender.

## References

[1] Chang SS, Baumgartner RG, Wells N, Cookson MS, Smith JA (2002). Causes of increased hospital stay after radical cystectomy in a clinical pathway setting. *Journal of Urology*.

[2] Chang SS, Cookson MS, Baumgartner RG, Wells N, Smith JA (2002). Analysis of early complications after radical cystectomy: results of a collaborative care pathway. *Journal of Urology*.

[3] Kanamaru S, Terai A, Ishitoya S (2004). Assessment of a protocol for prophylactic antibiotics to prevent perioperative infection in urological surgery: a preliminary study. *International Journal of Urology*.

[4] Takeyama K, Matsukawa M, Kunishima Y (2005). Incidence of and risk factors for surgical site infection in patients with radical cystectomy with urinary diversion. *Journal of Infection and Chemotherapy*.

[5] Hara N, Kitamura Y, Saito T, Komatsubara S, Nishiyama T, Takahashi K (2008). Perioperative antibiotics in radical cystectomy with ileal conduit urinary diversion: efficacy and risk of antimicrobial prophylaxis on the operation day alone. *International Journal of Urology*.

[6] Fujii T, Tabe Y, Yajima R (2011). Effects of subcutaneous drain for the prevention of incisional SSI in high-risk patients undergoing colorectal surgery. *International Journal of Colorectal Disease*.

[7] Benedetti Panici P, Zullo MA, Casalino B, Angioli R, Muzii L (2003). Subcutaneous drainage versus no drainage after minilaparotomy in gynecologic benign conditions: a randomized study. *The American Journal of Obstetrics and Gynecology*.

[8] Gallup DC, Gallup DG, Nolan TE (1996). Use of a subcutaneous closed drainage system and antibiotics in obese gynecologic patients. *The American Journal of Obstetrics and Gynecology*.

[9] Mangram AJ, Horan TC, Pearson ML, Silver LC, Jarvis WR (1999). Guideline for prevention of surgical site infection, 1999. Hospital Infection Control Practices Advisory Committee. *Infection Control and Hospital Epidemiology*.

[10] Matsumoto T, Kiyota H, Matsukawa M, Yasuda M, Arakawa S, Monden K (2007). Japanese guidelines for prevention of perioperative infections in urological field. *International Journal of Urology*.

[11] Kyoda Y, Takahashi S, Takeyama K, Masumori N, Tsukamoto T (2010). Decrease in incidence of surgical site infections in contemporary series of patients with radical cystectomy. *Journal of Infection and Chemotherapy*.

[12] van der Slegt J, van der Laan L, Veen EJ, Hendriks Y, Romme J, Kluytmans J (2013). Implementation of a bundle of care to reduce surgical site infections in patients undergoing vascular surgery. *PLoS ONE*.

[13] McGowan JE (1991). Cost and benefit of perioperative antimicrobial prophylaxis: methods for economic analysis. *Reviews of Infectious Diseases*.

[14] Baier PK, Glück NC, Baumgartner U, Adam U, Fischer A, Hopt UT (2010). Subcutaneous Redon drains do not reduce the incidence of surgical site infections after laparotomy. A randomized controlled trial on 200 patients. *International Journal of Colorectal Disease*.

[15] Magann EF, Chauhan SP, Rodts-Palenik S, Bufkin L, Martin JN, Morrison JC (2002). Subcutaneous stitch closure versus subcutaneous drain to prevent wound disruption after cesarean delivery: a randomized clinical trial. *The American Journal of Obstetrics and Gynecology*.

[16] Kaya E, Yetim I, Dervisoglu A, Sunbul M, Bek Y (2006). Risk factors for and effect of a one-year surveillance program on surgical site infection at a University Hospital in Turkey. *Surgical Infections*.

[17] Chelmow D, Rodriguez EJ, Sabatini MM (2004). Suture closure of subcutaneous fat and wound disruption after cesarean delivery: a meta-analysis. *Obstetrics and Gynecology*.

[18] Ramsey PS, White AM, Guinn DA (2005). Subcutaneous tissue reapproximation, alone or in combination with drain, in obese women undergoing cesarean delivery. *Obstetrics and Gynecology*.

[19] Zegers M, De Bruijne MC, Wagner C (2009). Adverse events and potentially preventable deaths in Dutch hospitals: results of a retrospective patient record review study. *Quality and Safety in Health Care*.

[20] Pronovost P, Needham D, Berenholtz S (2006). An intervention to decrease catheter-related bloodstream infections in the ICU. *New England Journal of Medicine*.

[21] Berenholtz SM, Pham JC, Thompson DA (2011). Collaborative cohort study of an intervention to reduce ventilator-associated pneumonia in the intensive care unit. *Infection Control and Hospital Epidemiology*.

